# Identification of Ligand-Receptor Pairs Associated With Tumour Characteristics in Clear Cell Renal Cell Carcinoma

**DOI:** 10.3389/fimmu.2022.874056

**Published:** 2022-06-06

**Authors:** Fahui Liu, Ping Wang, Wenjuan Sun, Yan Jiang, Qiming Gong

**Affiliations:** ^1^ Department of Medical Biochemistry and Cell Biology, Institute of Biomedicine, University of Gothenburg, Gothenburg, Sweden; ^2^ Department of Dermatology, Fujian Provincial Geriatric Hospital, Fuzhou, China; ^3^ Department of Internal Medicine, Chonnam National University Medical School, Gwangju, South Korea; ^4^ Guixi Key Laboratory for High Incidence Diseases, Youjiang Medical University for Nationalities, Baise, China; ^5^ Department of Nephrology, Affiliated Hospital of Youjiang Medical University for Nationalities, Baise, China

**Keywords:** ccRCC, ligand-receptor, single-cell, TCGA, tumor microenvironment

## Abstract

The tumour microenvironment (TME) of clear cell renal cell carcinoma (ccRCC) comprises multiple cell types, which promote tumour progression and modulate drug resistance and immune cell infiltrations *via* ligand-receptor (LR) interactions. However, the interactions, expression patterns, and clinical relevance of LR in the TME in ccRCC are insufficiently characterised. This study characterises the complex composition of the TME in ccRCC by analysing the single-cell sequencing (scRNA-seq) data of patients with ccRCC from the Gene expression omnibus database. On analysing the scRNA-seq data combined with the cancer genome atlas kidney renal clear cell carcinoma (TCGA-KIRC) dataset, 46 LR-pairs were identified that were significantly correlated and had prognostic values. Furthermore, a new molecular subtyping model was proposed based on these 46 LR-pairs. Molecular subtyping was performed in two ccRCC cohorts, revealing significant differences in prognosis between the subtypes of the two ccRCC cohorts. Different molecular subtypes exhibited different clinicopathological features, mutational, pathway, and immune signatures. Finally, the LR.score model that was constructed using ten essential LR-pairs that were identified based on LASSO Cox regression analysis revealed that the model could accurately predict the prognosis of patients with ccRCC. In addition, the differential expression of ten LR-pairs in tumour and normal cell lines was identified. Further functional experiments showed that CX3CL1 can exert anti-tumorigenic role in ccRCC cell line. Altogether, the effects of immunotherapy were connected to LR.scores, indicating that potential medications targeting these LR-pairs could contribute to the clinical benefit of immunotherapy. Therefore, this study identifies LR-pairs that could be effective biomarkers and predictors for molecular subtyping and immunotherapy effects in ccRCC. Targeting LR-pairs provides a new direction for immunotherapy regimens and prognostic evaluations in ccRCC.

## Introduction

Clear cell renal cell carcinoma (ccRCC) is the most common (70%–80%) histological subtype of kidney cancer with the worst prognosis (1). ccRCC exhibits a broad range of metastatic phenotypes, and patients with ccRCC displaying metastases have a 5-year survival rate between 10% and 20% (2). Various clinical trials using immune-based combinations for the treatment of metastatic ccRCC have shown long-term benefits (3). However, a comprehensive analysis of the effect of clinicopathological features on survival in patients treated with first-line immune checkpoint inhibitors and tyrosine kinase inhibitors for ccRCC can aid in clinical decision-making (4) as their efficacy remains limited (5–7). Few patients treated with immune-based combinations displayed unresponsive reactions and intrinsic or acquired resistance (8). A recent study showed that tumour and tumour-infiltrating cells are involved in drug resistance or unresponsiveness to cancer treatment strategies (9). However, the mechanisms underlying these phenomena remain unclear. Therefore, studying the intra-tumoral heterogeneity of cancer is vital to cancer research. ccRCC is highly heterogeneous, and varying patients show significant differences in the composition of the tumour and other cells within the tumour microenvironment (TME), affecting tumour progression and treatment resistance (10). Furthermore, intra-tumoral heterogeneity hinders accurate prognosis prediction and appropriate treatment development. Although the application of genetic signatures from the bulk RNA-seq data shows promise in identifying patient subgroups who respond to treatment, it provides a limited mechanistic understanding of the cell types responsible for regulating clinical benefit (11).

Conversely, single-cell RNA sequencing (scRNA-seq) enables a more comprehensive characterisation of cellular composition and transcriptional state, thereby providing insights into the transcriptional state of different cells and cell-cell interactions in the TME (12) and the impact of these events on disease progression and treatment (10). The TME consists of multiple cell types, including malignant, stromal, and immune cells (13). The heterogeneity of each cell type further increases the complexity and heterogeneity of the tumours, which eventually creates tumour cell or immune cell subpopulations (14). The different cell types in the TME communicate *via* ligand-receptor(LR) interactions, and this communication is associated with tumorigenesis, tumour progression, therapeutic resistance, immune cells infiltration and inflammation (15, 16). Therefore, it is crucial to understand the cell-cell interactions occurring within the TME and their effect on clinical outcomes to accurately determine risk stratification.

In this study, by combining the analysis of the single-cell dataset of ccRCC and high-throughput large scale sequencing data from TCGA-KIRC, the complex cell types in the TME of ccRCC are described. Additionally, a cellular regulatory network is constructed based on cell-cell communication analysis, and the potential clinical implications of cell-cell interactions in ccRCC are described. On further analysing LR-pairs in different cell types, two LR-pairs associated with molecular subtyping models were established. The molecular subtyping models were significantly associated with survival in both cohorts. Patients grouped using molecular subtyping showed different clinical-pathological characteristics, mutation characteristics, route characteristics, immunological characteristics, and immunotherapy response degrees.

## Methods and Materials

### Datasets

scRNA-seq data (GSE159115) of ccRCC was obtained from the Gene Expression Omnibus (GEO) database, which comprises 14 samples from nine patients with kidney cancer, including seven ‘ccRCC’ tumour samples, one ‘Chromophobe RCC’ tumour sample, and six adjacent normal samples. The seven ‘ccRCC’ tumour samples were included in subsequent analyses. The cell count of the GSE159115 primary tumour sample was obtained from gse159115.raw_cellCount.txt. 526 samples of ccRCC RNA-Seq data downloaded from the TCGA-KIRC portal were used as the training cohort. Additionally, the Research Concept and Research Activities- European Project (RECA-EU) dataset, comprising 91 ccRCC samples downloaded from the International Cancer Genome Consortium (ICGC) database, was used as an independent validation cohort.

### scRNA-seq Data Analysis

R (version 3.6.0) and the Seurat R package (version 3.6.3) were used for the analyses. Using the Seurat R package, Seurat objects were created for each sample with the cell-by-gene count matrix using CreateSeuratObject (arguments: min. cells = 5). Cells with high mitochondrial content (25% for tumour libraries) and low gene number detection (<300) were considered low-quality cells and discarded. Potential doublets identified *via* scrublet were also removed from further analyses. Subsequently, 20851 high-quality cells were obtained after quality checks. The relationship between the percentage of mitochondrial genes and mRNA reads was detected and visualised as the relationship between the number of mRNAs and reads of mRNA. Furthermore, all highly variable genes in single cells were identified after controlling for the average expression and dispersion relationship. Subsequently, principal component analysis with variable genes was used as the input to identify significant principal components (PCs) based on the jackStraw function. When different samples were pooled, highly variable genes were identified, and batch correction using canonical correlation analysis *via* Seurat was applied based on the highly variable genes to remove the batch effect before clustering. Cells were projected into a 2-D map with t-distributed stochastic neighbour embedding for visualisation. With a resolution of 0.2, cells were clustered using the ‘FindClusters’ function into 14 different cell types (clusters 0-13). The ‘FindAllMarkers’ function was used to identify differentially expressed genes (DEGs) in each cluster. Moreover, a few classical markers of cell subset definition were obtained from previous studies (17) and manually annotated according to marker expression.

### Cell-Cell Communication Analysis

Cell communication analysis was performed using CellPhoneDB (18). A permutation test calculated the significant mean and significance of cell communication based on cell interactions and the normalised cell matrix. LP-pairs were obtained for each cell pair with nominal p  <  0.05. Moreover, LR interactions are based on the annotations from the database included in the current study. At least one gene in the LR-pairs is the receptor. Additionally, receptor-receptor and other receptor-not-defined interactions were excluded.

### Correlation of LR-Pairs

The co-expression of a ligand and its corresponding receptor is essential for cell-cell communication. Therefore, Pearson’s correlation coefficients for the significant LR-pairs were calculated in the cell communication analysis using the TCGA-KIRC dataset. LR-pairs with Pearson’s correlation coefficient greater than 0.4 (p < 0.01) were used for consensus clustering analysis to identify molecular subtypes.

### Molecular Subtyping Based on LR-Pairs

Using the significantly relevant LP-pairs, the molecular subtypes of the samples were identified using consensus clustering in both the TCGA-KIRC and RECA-EU cohorts. ‘euclidean’ was chosen as the distance metric for the PAM algorithm, and 500 bootstrap replicates were performed, each of which included 80% of the training set. The number of clusters (k) was set from 2 to 10, and the best classification was determined by computing the consensus matrix and cumulative distribution function.

### Gene Set Enrichment Analysis and Functional Annotation

To study the biological pathways in different molecular subtypes, GSEA was used. The ‘hallmark’ gene set collection from the molecular signature database was used for pathway enrichment analysis. The clusterProfiler (19) package was used for functional annotation.

### Cell Culture and Transfection

Human ccRCC cell line 786-O (KCB200815YJ, Kunming, China) and renal epithelial cell HK-2 (KCB200941YJ, Kunming, China) were obtained from the Chinese Academy of Sciences. All cell lines were cultured in a DMEM medium containing 10% FBS. 786-O cells were infected with CX3CL1 lentivirus (Ubi-MCS-CBh-gcGFP-IRES-Puro-CX3CL1) (Shanghai Gene Chem Co., Ltd.) to against the CX3CL1 gene.

### RNA Analysis

Total RNA was extracted from 786-O and HK-2 cells using the TRIzol Reagent (Cowin Biosciences, Beijing, China) and converted into cDNA using Reverse Transcription Kit (Thermo Fisher Scientific, Waltham, USA). RT-qPCR was performed based on SYBR Green (Cowin Biosciences, Beijing, China) and an ABI 7500 instrument (Thermo Fisher Scientific, Inc.). The sequences of the primer pairs are listed in **Table S1**. RT-qPCR experiment was performed in triplicate.

### Cells Clone, Transwell Migration, Invasion, Western Blot and Immunofluorescence Assay

786-O cells were plated onto the upper 8μm transwell chamber (corning, USA) per-coated with Matrigel (Corning, USA). Cells were fixed with paraformaldehyde and stained with 0.1% crystal violet solution to perform Transwell Assay. For Colony Assay, 786-O cells were re-seeded onto 24-well plates at a density of 100 cells per well and stained with 0.1% crystal violet solutions after 4% paraformaldehyde solution was fixed. For Western Blot and Immunofluorescence, cells were incubated with anti-CX3CL1(Cat# DF12376, Affinity Biosciences), anti-β-Tubulin (Cat#T0023, Affinity Biosciences) and anti-GAPDH (Cat# T0004, Affinity Biosciences) as described by previous study (20).

### Analysis of Immune Infiltration in ccRCC

The 22 immune-cell proportions for each sample and immune cell subsets were inferred using the CIBERSORT algorithm (21) with the LM22 gene set. Meanwhile, the scores for stromal cells and immune cell infiltration levels in ccRCC tumour tissues were calculated using the ESTIMATE algorithm.

### Risk Model

The risk score for each patient was calculated using the following formula: LR.Score = ∑beta_i_ × Exp_i_, where i indicates the expression level of LR-pairs and beta indicates the coefficient of the LR-pairs of multivariate Cox regression. Based on the threshold of 0, patients were divided into high and low-score groups, and the survival curve was plotted using Kaplan–Meier analysis.

### Statistical Analysis

GraphPad Prism 8.0 software was used for data analysis. All data were computed as the means ± standard (SD) deviation under three independent experiments. The significance of two group differences was analyzed with Student’s t-test. P<0.05 was statistically significant.

## Result

### Single-Cell Transcriptome Landscape of ccRCC

**Figure 1:**
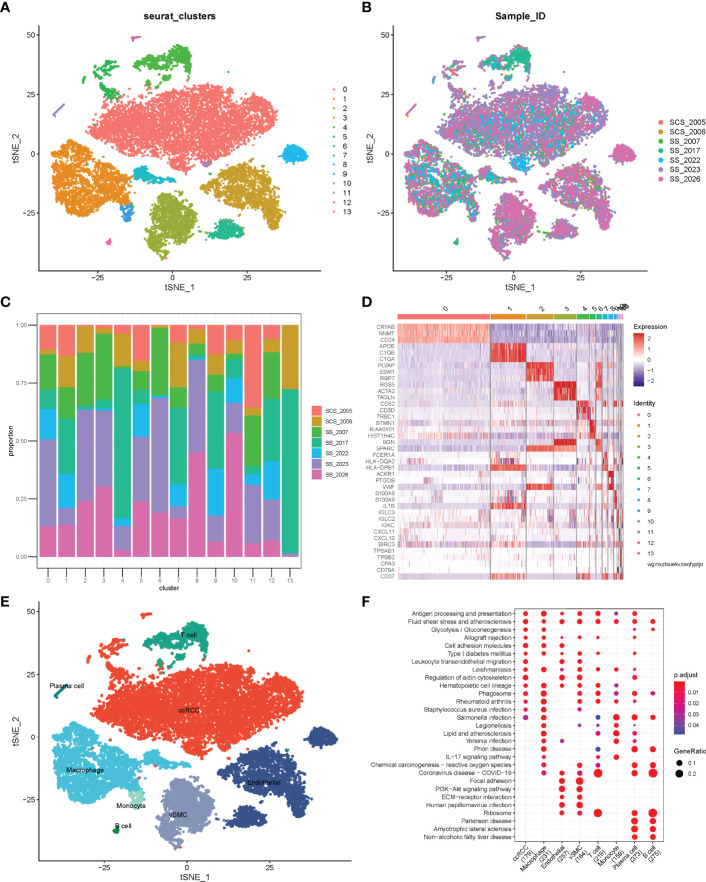
Single-cell atlas of ccRCC tissues. **(A)** tSNE of 20851 cells of seven clear cell renal cell carcinoma (ccRCC) samples. **(B)** Distribution of cells from different samples; **(C)** Stacked bar plots showing the frequencies of 14 cell types in seven samples. **(D)** Heatmap of the top three marker genes for different clusters. **(E)** tSNE demonstrating different cell types in ccRCC. **(F)** Kyoto Encyclopedia of Genes and Genomes pathway enrichment analysis of cell subset marker genes.

Before quality control, correlations between unique molecular identifier (UMI) numbers, mitochondrial genes and mRNA numbers were analysed (**Figure S1A, B**), which revealed that UMI number was not significantly correlated with mitochondrial gene percentage (**Figure S1A**) but was positively correlated with mRNA number (**Figure S1B**). Additionally, the number of mRNAs, the readings of mRNA and the distribution of mitochondrial and nuclear chromosome genes were also analysed (**Figure S1C**). Most gene numbers showed a distribution around 0–8000 while the mitochondrial percentage was below 25%. Further, cells with more than 25% mitochondrial genes and fewer than 300 genes were deleted. Potential doublets were also predicted *via* ‘scrublets,’ which were removed in subsequent analyses. The gene expression profile of 20851 cells was obtained, and the number of cells in each sample was counted (**Table 1**). The number of filtered mRNAs, the readings of mRNA and the distribution of mitochondrial and nuclear chromosome genes are shown in **Figure S1D**. Highly variable genes (the first 3000) were shortlisted after quality control for further downstream analysis (**Figure S1E**). Cell features were extracted using principal component analysis, identifying 14 clusters (**Figure 1A, B**, cell_cluster.txt), with cluster 0 being derived from patient SCS_2023 and cluster 10 from patient SCS_2026 (**Figure 1C**). The identification of DEGs in each cluster was conducted using Wilcoxon rank and testing, and the top 3 DEGs in each cluster were labelled using a heat map (**Figure 1D**). Finally, 14 clusters were annotated to eight cell types based on classical markers (**Table 2**). Further, the Kyoto Encyclopedia of Genes and Genomes pathway enrichment analysis of marker genes for different cellular subsets was performed (**Figure 1F**), revealing that different cellular subsets share common pathways, such as Antigen processing and presentation, Fluid shear stress and atherosclerosis pathways.

**Table 1:** Statistical analysis of cell numbers for seven clear cell renal cell carcinoma (ccRCC) samples.

Sample	raw_cellCount	clean_cellCount	percent
SCS_2005	1704	1673	0.981807512
SCS_2006	1660	1374	0.827710843
SS_2007	3674	3345	0.910451824
SS_2017	2872	2817	0.980849582
SS_2022	1953	1942	0.99436764
SS_2023	6453	6140	0.951495428
SS_2026	3635	3560	0.979367263

**Table 2:** Annotation of cell types for 14 cell clusters.

Cell Type	Cluster	Number of Cells
clear cell renal cell carcinoma (ccRCC)	0	8819
Macrophage	1	3382
Endothelial	2	2584
vascular smooth muscle cells (vSMC)	3	2169
T cell	4	1176
ccRCC	5	573
Endothelial	6	534
Macrophage	7	502
Endothelial	8	498
Monocyte	9	248
Plasma cell	10	122
ccRCC	11	87
ccRCC	12	85
B cell	13	72

### Complex Intercellular Communication Networks in ccRCC

**Figure 2:**
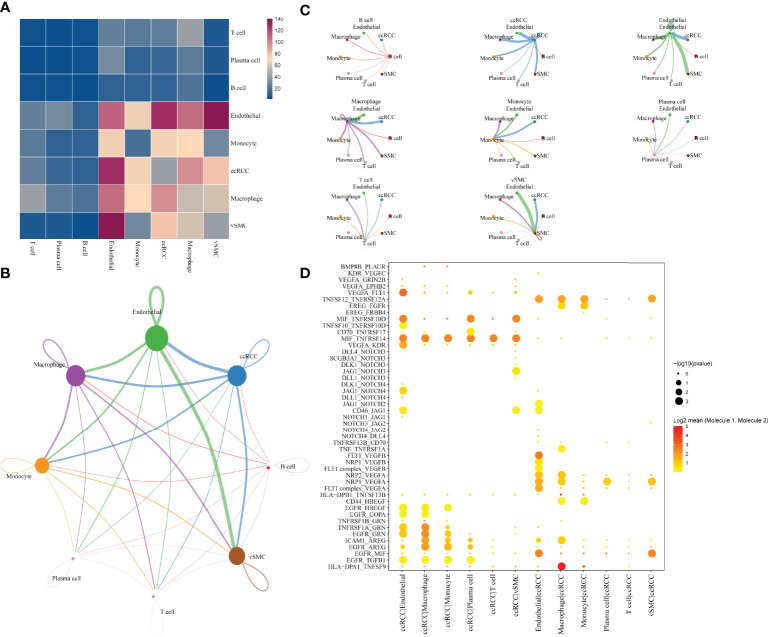
Communication and networking between cells in ccRCC. **(A)** Network of the number of significant interaction events between different cellular subsets. **(B)** Overview of selected statistically significant interactions between clear cell renal cell carcinoma, macrophages and other cell types. **(C)** Detailed network of cell-cell interactions among eight cell subsets. **(D)** tp Intensity and specificity of ligand-receptor pairs in different cell types.

The single-cell analysis identified eight cell types in the TME. To further investigate the potential interactions between different cell types in the TME of ccRCC, cell-to-cell interactions were analysed using cellphoneDB, wherein ccRCC was revealed to have many interactions with other cell subsets, showing the highest interaction strength with endothelial and macrophage cells. Additionally, endothelial cells had a strong interaction with vascular smooth muscle cells(vSMC) (**Figure 2A**). The interaction network between the eight cell subsets aids in visualising the many interactions within and between the cell subsets (**Figure 2B**), with thicker lines and larger nodes indicating more significant LR between the cell subsets. Moreover, the cell subsets of ccRCC, macrophages and vSMC showed the most cell-to-cell interactions within and between the cell subsets (**Figure 2C**). Genes in the Hedgehog, Notch, TGFβ, WNT signalling and EGFR signalling that are related to tumour proliferation, metastasis and progression were selected to further determine the presence of a significant interaction between the cell subsets. The result shows many interactions between the receptor HLA−DPA1 and its corresponding ligand TNFSF9 in the cell subset of macrophage and ccRCC while EGFR and MIF have strong interactions between different cell subsets (endothelia, ccRCC, vSMC) (**Figure 2D**).

### Construction of Molecular Subtypes Based on LR-Pairs

**Figure 3:**
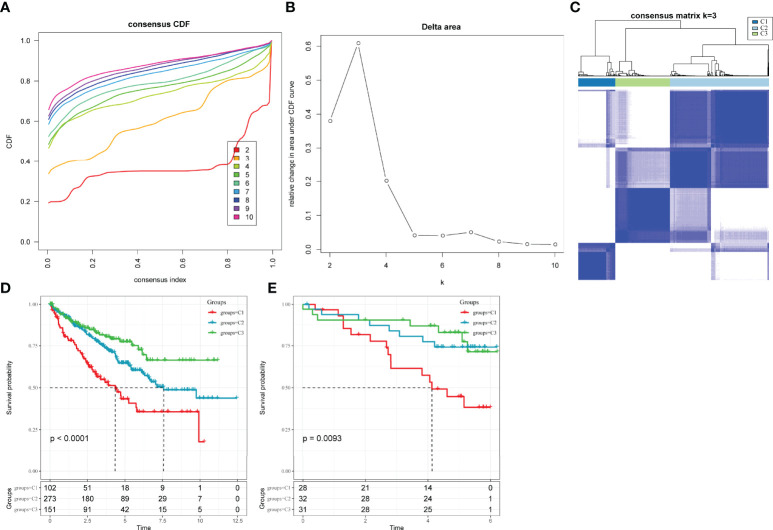
Molecular subclassification of ccRCC in different cohorts. **(A)** CDF curves for k = 2–10 in the TCGA-KIRC cohort. **(B)** CDF delta area curve in the TCGA-KIRC cohort. **(C)** Heatmap clustering of TCGA-KIRC datasets when consensus (k) = 3. **(D)** The difference in survival among different molecular subtypes in the TCGA-KIRC cohort. **(E)** The difference in survival among different molecular subtypes in the Research Concept and Research Activities- European Project cohort.

LR interactions between different cell types in the TME play a vital role in the occurrence and development of tumours. Thus, LR-pairs that interact significantly in different cell types were extracted based on Pearson’s correlation coefficient between ligand and receptor expressions. A total of 126 LR-pairs that were significantly correlated in TCGA-KIRC were identified (tcga.LR.cor.res.txt). Furthermore, the expression level of LR-pairs was determined by the sum of the expression values of the receptor and ligand genes in TCGA-KIRC. A total of 46 LR-pairs that were significantly associated with patient prognosis in TCGA-KIRC (p < 0.01) (tcga.LR.HR.res.txt) were used in molecular subtyping analysis. A total of 526 ccRCC samples in the TCGA-KIRC cohort were clustered using ConsensusClusterPlus. The optimal number of clusters is determined by the cumulative distribution function and delta area curve (**Figures 3A, B**), and k = 3 was selected to obtain three molecular subtypes (**Figure 3C**; tcga.subtype.txt) which showed significant differences in prognosis (p < 0.0001)(**Figure 3D**). On comparing the three subtypes, the C3 subtype had a better prognosis, while the C1 subtype had a worse prognosis. Similarly, patients with ccRCC in the RECA-EU cohort were typed (icgc.subtype.txt). The results showed that there were significant differences in prognosis among the three molecular subtypes (p = 0.0093) (**Figure 3E**), which was consistent with the training set.

### Comparison of Different Molecular Subgroups With Clinical Features

**Figure 4:**
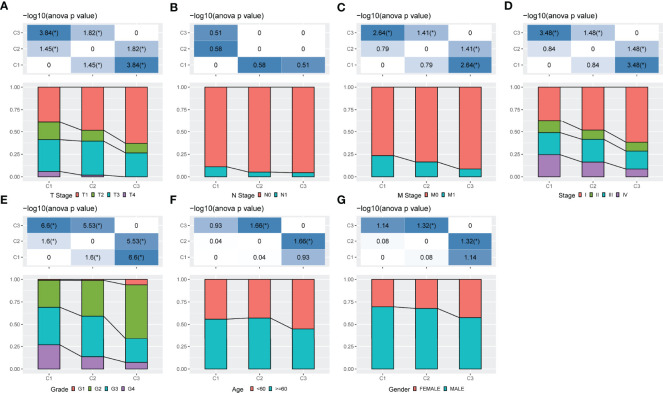
Comparison of different molecular subgroups with clinical information. **(A–G)** The distribution of samples in different groups in the TCGA-KIRC cohort. The horizontal axis represents the different groupings of samples while the vertical axis represents the percentage of clinical information in the corresponding grouped sample. Different colours represent different clinical information. The table above represents a certain clinical feature distribution in any two groups and analyses the p value using the chi-square test.

The distribution of clinical features in the three molecular subtypes was compared using the TCGA-KIRC cohort. The results showed that patients with poor prognosis in the C1 subtype had higher TNM stages, with significant differences between the C1 and C3 subtypes in terms of T and M stage distribution (p < 0.05) (**Figures 4A–C**). In the C1 and C2 subtypes, patients with Stage III and Stage IV ccRCC were higher in number than those in the C3 subtype (p < 0.05) (**Figure 4D**). A significant difference was also observed in Grade between different subtypes (p < 0.05) (**Figure 4E**). Additionally, significant differences in age and gender were observed among different subtypes (**Figures 4F, G**) (p < 0.05).

### Mutational Characteristics of Different Molecular Subtypes

**Figure 5:**
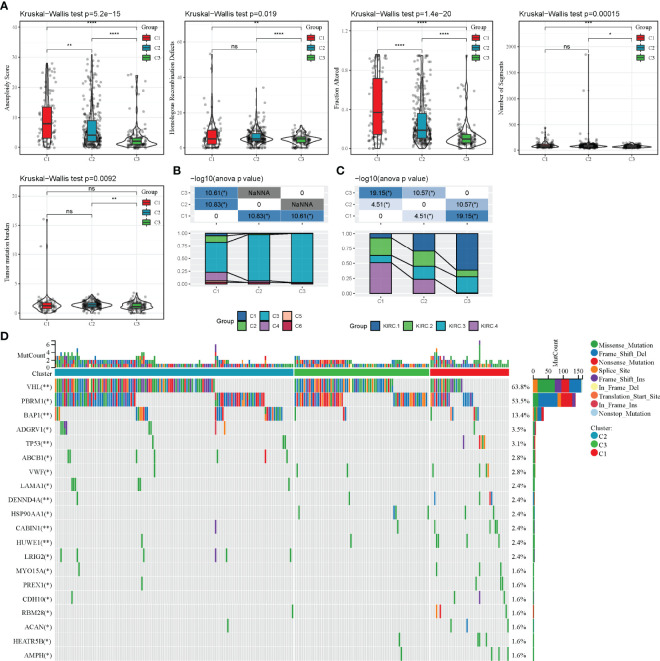
Mutation characteristics of different molecular subtypes. **(A)**: Difference analysis of homologous recombination defects, aneuploidy score, fraction altered genome, segment numbers and tumour mutation burden in the TCGA-KIRC cohort; **(C)**: Comparison of somatic mutation variation analysis in three molecular subtypes. nsp ≥ 0.05; *p < 0.05; **p < 0.01; ***p < 0.001; ****p < 0.0001.

The differences in genomic alterations of these three molecular subtypes in the TCGA cohort were further explored. The C1 and C2 subtypes showed higher levels of aneuploidy (p = 5.2e-15), homologous recombination defects (p = 0.019), fraction altered genome (p = 1.4e-20), segment numbers (p = 0.00015) and tumour mutation burdens (p = 0.0093) (**Figure 5A**). Additionally, information on the immune molecular subtypes of TCGA-KIRC was obtained from a previous study (22). The relationship between the six immune subtypes and three molecular subtypes defined in this study was compared. Between the C3 subtypes, the C3 immune subtype accounted for a more significant proportion. Among the C1 subtypes, the C1, C2, C4 and C6 immune subtypes accounted for a more significant proportion (**Figure 5B**). Furthermore, four additional subtypes (KIRC-C1, C2, C3, C4), which were obtained from a previous study (23), were compared with the three molecular subtypes defined in this study. The results showed that KIRC-C4 accounted for more significant proportion in the C1 molecular subtype, while KIRC-C1 accounted for the most proportion in the C3 molecular subtype (**Figure 5C**). Finally, the analysis of the correlation between gene mutations, copy number variants and molecular subtypes revealed a significant correlation between subtypes and gene mutations. The mutation frequencies of VHL, PBRM1 and BAP1 genes vary significantly among subtypes, with more mutation frequencies observed in the C1 subtype (**Figure 5D**).

### Pathway Analysis of Different Molecular Subtypes

**Figure 6:**
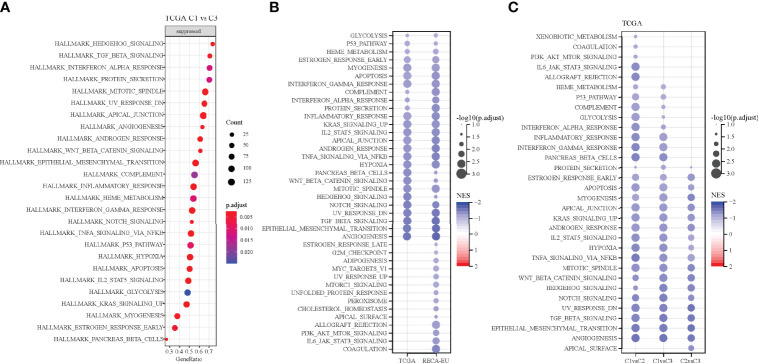
Pathway analysis of different molecular subtypes. **(A)** Analysis of signalling pathways for differentially expressed genes in the C1 vs. C3 subtypes of the TCGA-KIRC cohort. **(B)** Pathway analysis of the differentially expressed genes in the C1 vs. C3 subtypes in two clear cell renal cell carcinoma cohorts. **(C)** Gene set enrichment analysis of the comparison between different molecular subtypes.

Pathways that are differentiated in different molecular subtypes were analysed using GSEA. The results showed the significant enrichment of 26 pathways in the C1 subtype compared with the C3 subtype in the TCGA cohort and 40 pathways in the RECA-EU cohort (**Figures 6A, B**). Overall, the inhibition pathways contain few immune marker pathways, such as INTERFERON_GAMMA_RESPONSE, COMPLEMENT, INTERFERON_ALPHA_RESPONSE and INFLAMMATORY_RESPONSE. (**Figure 6A**). Additionally, abnormal pathways between the C1 and C3 subtypes in different ccRCC cohorts are shown in **Figure 6B**. The pathways for TCGA-KIRC cohorts between C1 and C2 subtypes, C1 and C3 subtypes and C2 and C3 subtypes are shown in **Figure 6C**. Overall, immunomodulatory pathways in patients with the C1 subtype are inhibited.

### Immune Characteristics of Different Molecular Subtypes

**Figure 7:**
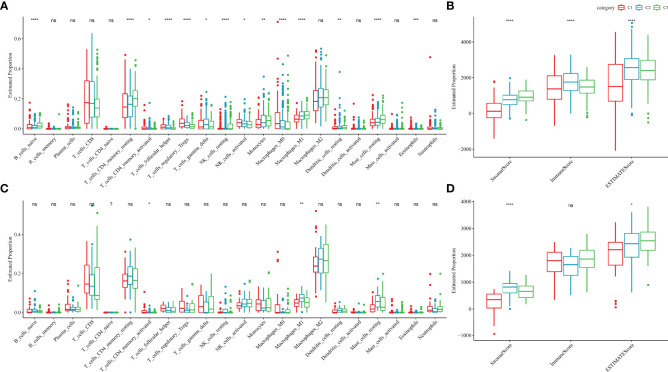
Immune characteristics of different molecular subtypes. **(A, B)** Difference analysis of immune cell scores in the TCGA-KIRC cohort calculated using the CIBERSORT and ESTIMATE algorithms. **(C, D)** Difference analysis of immune cell scores in the Research Concept and Research Activities- European Project cohort calculated using the CIBERSORT and ESTIMATE algorithms. nsp ≥ 0.05; *p < 0.05; **p<0.01; ***p<0.001; ****p<0.0001.

To further elucidate the differences in the immune microenvironment of patients in different molecular subtypes, the degree of infiltration of 22 immune cells in the two ccRCC cohorts was assessed using the CIBERSORT algorithm. In both cohorts, resting mast cells, M1 macrophages and activated/memory CD4^+^ T cells showed significant differences in different molecular subtypes (**Figures 7A, C**). ESTIMATE algorithm was also used to assess immune cell infiltration in each sample. The ‘ImmuneScore’ of the C3 subtype in the TCGA and RECA-EU cohorts was higher than the C1 subtype, indicating that the C3 subtype has a high immune cell infiltration degree (**Figure 7B, D**).

### Model Construction Based on the LR-Pairs Score

The above analyses show that the LR-pair molecular subtypes have different mutation landscapes, pathway characteristics and immune infiltration degrees. A total of 46 LR-pairs were significantly associated with patient prognosis, with 37 of them significantly different in both the TCGA-KIRC and RECA-EU datasets (all.LR.genes.diff.kruskal.res.txt) (FDR < 0.001). The 37 LR-pairs with significant differences were further compressed using LASSO cox regression in the TCGA-KIRC cohort to reduce the number of genes in the risk model. The trajectory of each independent variable is shown in **Figure S2A**, wherein with the gradual increase of lambda, the number of independent coefficients tending to 0 also gradually increases. Model performance was evaluated using 10-fold cross-validation.

**Figure 8:**
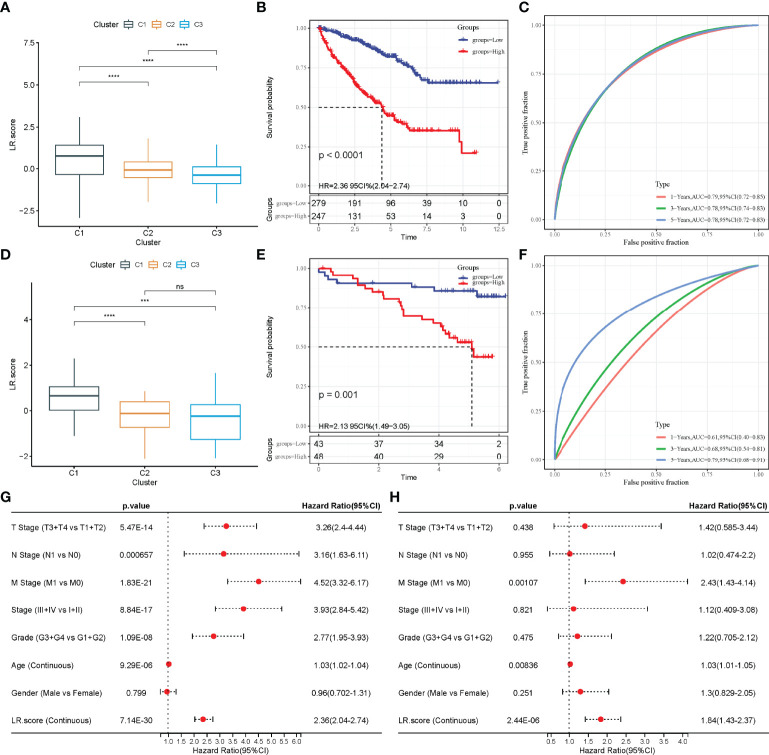
Construction of LR.score model. **(A)** The difference in LR.score between the different molecular subtype groups in the TCGA-KIRC cohort. **(B)** Survival benefit of LR.score in the high and low LR.score groups in the TCGA-KIRC cohort. **(C)** The predictive value of LR.score in patients among the TCGA-KIRC cohort. **(D)** The difference of LR.score between the different molecular subtype groups in the Research Concept and Research Activities- European Project (RECA-EU) cohort. **(E)** Survival benefit of LR.score in the high and low LR.score groups in the RECA-EU cohort. **(F)** The predictive value of LR.score in patients among the RECA-EU cohort. **(G)** Univariate cox regression analysis of LR.score, age, gender, TNM stage and grade for overall survival (OS) in the TCGA-KIRC cohort. **(H)** Multivariate cox regression analysis of LR.score, age, gender, TNM stage and grade for OS in the TCGA-KIRC cohort; ns, p≥ 0.05; ***p<0.001; ****p<0.0001.

On analysing the confidence interval under each lambda (**Figure S2B**), the model was found to be optimal when lambda = 0.0137, and 12 LR-pairs at lambda = 0.0137 were selected for further analysis. Furthermore, the model was optimized using stepwise multivariate regression analysis. Finally, 10 LR-pairs, including ‘APLNR_APLN’, ‘CSF1R_CSF1’, ‘CX3CR1_CX3CL1’, ‘EPHA4_EFNB3’, ‘FGFR3_EPHA4’, ‘HGF_CD44’, ‘KDR_VEGFC’ ‘NGF_NGFR,’ ‘TEK_ANGPT1’ and ‘TEK_ANGPT4’ were identified as crucial LR-pairs. The multivariate Cox regression coefficient results for these 10 LR-pairs are shown in **Figure S2C**. Furthermore, the LR-pairs scoring model was constructed using the 10 LR-pairs to facilitate the quantitative analysis of LR-pair scores in patients with ccRCC. The results showed that the LR.score in the C1 subtypes was significantly higher than that in the C2 and C3 subtypes (**Figure 8A**). To further assess the clinical relevance of LR-pair scores, patients were defined as high LR.score group if the LR.score > 0 and low LR.score group otherwise. Patients with low LR.score in the TCGA-KIRC cohort had a better prognosis than patients with high LR.score (p < 0.0001) (**Figure 8B**). The area under the curve (AUC) of the time-dependent receiver operating characteristic (ROC) curves of the LR.score was 0.79, 0.78 and 0.78 at 1, 3 and 5 years, respectively (**Figure 8C**). Univariate and multivariate cox regression analyses were also used in analysing the TCGA-KIRC cohort, which showed that LR.score is a reliable and independent prognostic biomarker for assessing the prognosis of patients with ccRCC (**Figures 8G, H**). Furthermore, the reliability of the LR.score was validated using 91 samples from the RECA-EU cohort. A study about LR.score in different molecular subtypes reported similar conclusions using the RECA-EU cohort (**Figure 8D**). Patients with low LR.score in the RECA-EU dataset showed a significant survival benefit (p = 0.001) (**Figure 8E**). The AUC of the time-dependent ROC curves of the LR.score was 0.61, 0.68 and 0.79 at 1, 3 and 5 years, respectively (**Figure 8F**).

### Differences in LR.score in Different Clinical Subgroups

**Figure 9:**
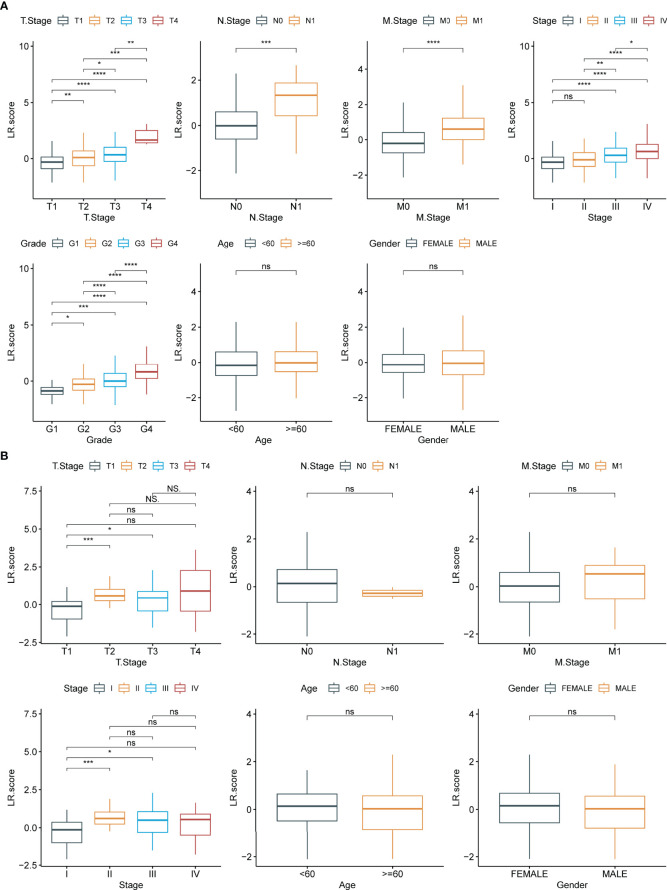
Clinical features of LR.score. **(A)** Differences in LR.score in different clinical subgroups in the TCGA-KIRC cohort. **(B)** Differences in LR.score in different clinical subgroups in the Research Concept and Research Activities- European Project cohort. nsp≥0.05; *p<0.05; **p<0.01; ***p<0.001; ****p<0.0001.

To examine the relationship between LR.score and clinical features of ccRCC, the differences in LR.score between subgroups of different clinical features in the TCGA-KIRC dataset were compared. Patients showed significant differences in TNM stages, pathological stages and histological grades when compared with LR.score. LR.score varied significantly between different clinical feature subgroups, with high malignancy degree correlating to high LR.score (**Figure 9A**). Additionally, the relationship between the LR.score and clinical-pathological features in RECA-EU was analysed. The results showed that the differences in the different subgroups of LR.score were not apparent, possibly due to the insufficient sample size in the RECA-EU cohort (**Figure 9B**).

### Correlation Between LR.score and Immune-Related Features

**Figure 10:**
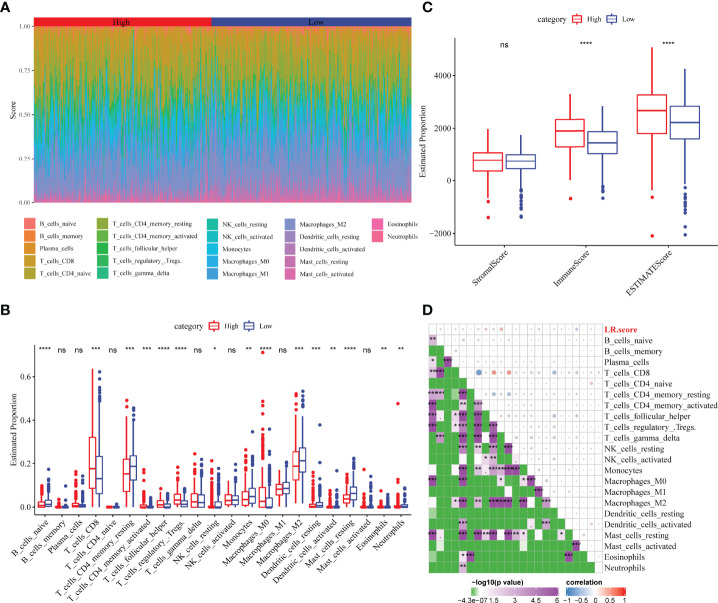
Correlation between LR.score and immune-related characteristics. **(A)** Distribution and expression of the 22 types of immune cells in the TCGA-KIRC cohort. **(B)** Analysis of the immune cell scores between the different LR.score groups using the CIBERSORT algorithm. **(C)** Analysis of the immune cell scores between the different LR.score groups using the ESTIMATE algorithm. **(D)** Correlation between LR.score and immune cell score. ns p≥0.05; *p< 0.05; **p<0.01; ***p<0.001; ****p<0.0001.

The distribution of 22 immune cells in the TCGA-KIRC cohort and the differences between the LR.score groups were analysed. Significant differences in immune cell infiltration levels were observed between patients (**Figure 10A**). Additionally, immune cell infiltration levels showed significant differences between different LR.score groups. Further, CD8^+^ T cells exhibited a high level of infiltration, with the high LR.score group showing significantly higher infiltration levels than the low LR.score group (**Figure 10B**). Additionally, immune infiltration levels were also compared for different LR.score groups using the ESTIMATE algorithm, wherein the Immune score and ESTIMATE score in the high LR.score group were significantly higher than those in the low LR.score group (**Figure 10C**). Further, the correlation between LR.score and 22 immune cell scores in the TCGA-KIRC cohort was analysed using Pearson’s correlation coefficient. The result shows that LR.score was significantly positively correlated with activated/memory CD4^+^ T cells, follicular helper T cells and regulatory T cells but negatively correlated with resting mast cells (**Figure 10D**).

### The Relationship Between LR.score and Immunotherapy

**Figure 11:**
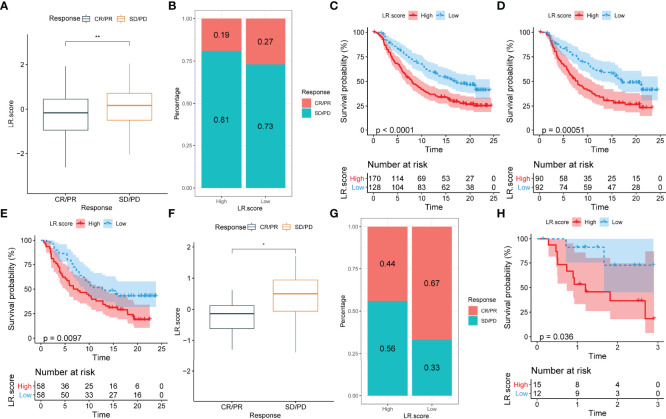
Correlation between LR.score and response to anti-PD-L1 immunotherapy. **(A)** Differences in LR.score between responders and non-responders in the IMvigor210 cohort. **(B)** The proportion of patients responding to immunotherapy in the high and low LR.score groups in the IMvigor210 cohort. **(C)** Prognostic differences between the high and low LR.score groups in the IMvigor210 cohort. **(D)** Prognostic differences between the high and low LR.score groups in early-stage patients in the IMvigor210 cohort. **(E)** Prognostic differences between the high and low LR.score groups in advanced patients in the IMvigor210 cohort. **(F)** LR.score differences between responders and non-responders in GSE78220. **(G)** The proportion of patients responding to immunotherapy in the high and low LR.score groups in GSE78220. **(H)** Prognostic differences between the high and low LR.score groups in GSE78220. ns p≥0.05; *p< 0.05; **p<0.01.

To identify the relationship between LR.score and immunotherapy, the value of LR.score to predict a patient’s response to immune checkpoint blockade(ICB) treatment was examined. In the anti-PD-L1 cohort (IMvigor210 cohort), 348 patients exhibited varying degrees of response to anti-PD-L1 receptor blockers, including complete response (CR), partial response (PR), stable disease (SD) and progressive disease (PD). Patients with SD/PD had a higher LR.score than patients with CR/PR (**Figure 11A**). Percentage statistics between the high and low LR.score groups showed significantly better treatment outcomes in patients with low LR.score (**Figure 11B**). Survival analyses further showed that LR.score was associated with overall survival in patients receiving immunotherapy (p < 0.0001) (**Figure 11C**). In early-stage patients receiving immunotherapy, LR.score was associated with overall survival (p = 0.000051) (**Figure 11D**) while in advanced patients it was also associated with overall survival (p = 0097) (**Figure 11E**).

Additionally, in another cohort of anti-PD1 (GSE78220), patients with SD/PD patients showed higher LR.score than those with CR/PR (**Figure 11F**). Moreover, percentage statistics between the high and low LR.score groups also showed that patients with low LR.score had significantly better treatment outcomes (**Figure 11G**), clinical benefit and prolonged overall survival (p = 0.036) (**Figure 11H**).

### CX3CL1 Knockdown Accelerates Migration and Invasion of ccRCC Cell *In Vitro*

**Figure 12:**
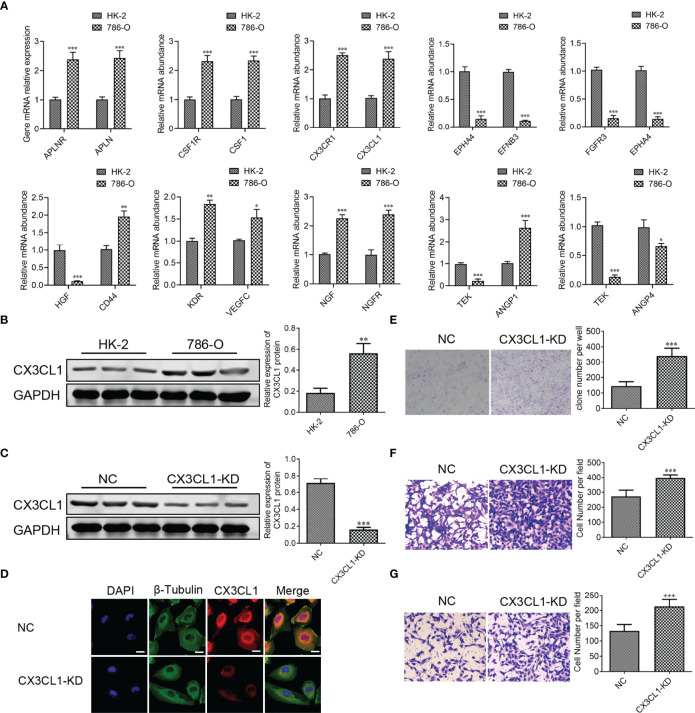
CX3CL1 Knockdown promoted migration and invasion of ccRCC cell *in vitro*. **(A)** Real-time RT-PCR assay of 10 LR-pairs mRNA expression levels in human ccRCC cell line 786-O and renal epithelial cell HK-2. **(B, C)** the protein expression of CX3CL1 was performed by Western Blot assay. **(D)** The subcellular localization of CX3CL1 was identified by immunostaining assay. Scale bar: 10μm. **(E)** Cell colonies of 786-O cell in normal control and CX3CL1-KD group. **(F, G)** 786-O cell migration assay **(F)** and invasion assay **(G)** were performed in normal control and CX3CL1-KD group. ns, p≥0.05; *p< 0.05; **p<0.01; ***p<0.001.

To further confirm the results of databases, 10 LR-pairs, including ‘APLNR_APLN,’ ‘CSF1R_CSF1’, ‘CX3CR1_CX3CL1’, ‘EPHA4_EFNB3’, ‘FGFR3_EPHA4’, ‘HGF_CD44’, ‘KDR_VEGFC,’ ‘NGF_NGFR,’ ‘TEK_ANGPT1’ and ‘TEK_ANGPT4’ were subjected to RT-qPCR assay in ccRCC cell line. As shown in **Figure 12A**, the mRNA levels of APLNR, APLN, CSF1R, CSF1, CX3CR1, CX3CL1, CD44, KDR, VEGFC, NGF, NGFR, ANGPT1 in the 786-O cell were up-regulated compared with normal renal epithelial cell HK-2, whereas the mRNA levels of EPHA4, EFNB3, FGFR3, HGF, ANGPT4, TEK in the 786-O cell were down-regulated compared with HK-2 cell. Recently some research found one such chemokine that plays a critical role in the anti-cancer procession is CX3C chemokine ligand 1 (CX3CL1) and its receptor CX3C chemokine receptor 1 (CX3CR1). In **Figure 12B**, we found that the protein expression of CX3CL1 was increased in the 786-O cell compared with the HK-2 cell. To demonstrate the specific role of CX3CL1, we knocked down the CX3CL1 gene in the 786-O cell (**Figures 12C, D**). We found that the numbers of clone formation (**Figure 12D**), as well as the migration (**Figure 12E**) and invasion capacities (**Figure 12F**), were notably increased in 786-O cells with CX3CL1 knockdown.

## Discussion

scRNA-seq approaches are rapidly being employed to describe the quantity and functional status of tumour-associated cell types in the TME, revealing previously unknown data about cellular heterogeneity (24). However, in addition to characterising the cellular makeup of tumours, it is crucial to understand how different cell types in the TME interact to initiate tumour progression (25). Although there exist studies examining cell-to-cell communication using bulk-seq and scRNA-seq data, investigations relating these features to biological outcomes and studies elucidating the significance of these interactions with specific clinical outcomes remain scarce. This study integrated bulk-Seq and scRNA-Seq data of ccRCC for analyses. After examining cell-to-cell communication, few critical LR-pairs were obtained, revealing cell complexity in the TME. Additionally, two molecular subtyping models were constructed based on these LR-pairs, and the prognostic evaluation and immunotherapy utility of different molecular subtyping was found. These findings aid in better understanding the role of cell-cell communication in the TME of ccRCC and developing novel therapy options for ccRCC.

Accumulating evidence shows that immune cell dysfunction within KIRC-TME induces immunosuppression and plays a critical role in tumour growth and treatment (25). In this study, eight different cell types were identified in the ccRCC’s TME, indicating its compositional complexity. Malignant solid tumour tissues contain not only tumour cells but also normal epithelial, stromal, immune and vascular cells, with stromal and immune cells as the most prominent components (26). Stromal cells play an essential role in tumorigenesis and drug resistance (27), while infiltrating immune cells are specific to certain environments (28). For example, T cell that infiltrates tumours has been demonstrated to have an anti-cancer effect in ovarian cancer, whereas it is associated with tumour growth, invasion and metastasis in colorectal cancer (28). These cells can impact the results of genomic analysis of tumour samples (such as expression profiles or copy numbers), thus, understanding the TME and the interactions between tumours and other cells could provide important insights into tumour biology and help build reliable prognostic, predictive models.

Therefore, to further study the interaction between different cell types in the TME of ccRCC, a comprehensive and systematic analysis of cell communication in the TME of ccRCC was conducted. Previous studies have shown that the interaction of ligands and receptors differs significantly in different types of tumours. These differences can lead to the activation or inhibition of different pathways, resulting in tumour development and drug resistance. Therefore, based on the significantly different LR-pairs obtained in this study, a new molecular subtyping model was constructed. According to the LR-pairs model, patients in different KIRC cohorts can be effectively divided into three subtypes, and the prognosis of patients with different molecular subtypes is significantly different. Additionally, varying clinical-pathological features, mutation features, pathways and immune features showed significant differences in different molecular subtypes.

Furthermore, to further confirm the effectiveness of our typing analysis, data on the immune subtypes of TCGA-KIRC were obtained from a previous pan-cancer study, wherein ccRCC samples were divided into six molecular subtypes based on 160 different immune gene signatures (22). This study compared the relationship between these six immune subtypes and the three LR-pairs subtypes defined in the current study. The results showed that the C3 immune subtypes obtained from the previous study accounted for more of the C3 molecular subtype defined in this study. In previous studies, the C3 immune subtype was described as an ‘inflammatory’ subtype. Furthermore, the C3 immune subtype was distinguished from other subtypes by increased Th17 and Th1 gene expression, low to medium cancer cell proliferation and lower levels of aneuploidy and total somatic copy number changes. Additionally, it showed the best prognosis among these six immune molecular subtypes, which is consistent with the best outcome of the C3 molecular subtype defined in the current study. Moreover, the immune subtypes C1, C2, C4 and C6 with worse prognosis accounted for more than the C1 molecular subtypes defined in this study, which coincides with the poor prognosis of C1.

Additional molecular subtypes were provided in previous studies, and four molecular subtypes (KIRC-C1, C2, C3, C4) were identified *via* consensus clustering (23). The KIRC-C3 subtype was associated with the worst prognosis while the KIRC-C1 subtype had the best prognosis. The relationship between these four molecular subtypes and our three molecular subtypes was compared, wherein the KIRC-C4 subtype accounted for more than the C1 subtype, while the KIRC-C1 subtype accounted for the most significant proportion of the C3 subtype. Consistent with previous studies, the reliability of the present study is reinforced, providing a base for further understanding of the interactions between cells in the TME and developing new molecular typing methods for patients with ccRCC.

Currently, individualised models based on the specific biomarkers of tumour subtypes have been established in breast and colorectal cancers to improve patients’ prognoses (29, 30). However, clinically efficient individualised models for patients with ccRCC are scarce. Considering the individual heterogeneity of the TME, it is urgent to quantify the scoring pattern of individual tumours and establish an effective treatment and prognosis evaluation model for patients with ccRCC. Although previous studies have explored the value of different signatures in the prognostic evaluation of ccRCC, their validity remains limited (31, 32). Meanwhile, the analysis of cell-cell communication reveals that the expression of ligands and receptors and the type of interaction vary in different tumour types. Therefore, focusing on the communication of different cell types in the TME and their interactions could aid in diagnoses and treatment options. In the current study, based on the previous analysis of LR-pairs with significant differences in both the TCGA-KIRC and RECA-EU datasets, 10 LR-pairs were identified as potential ‘subtype biomarkers’. The LR-pairs scores model was established to quantify different risk scores among individuals.

CX3CL1 is a chemokine with a unique motif -Cys-X-X-X-Cys- at the N-terminal end structure and the only member of the δ-chemokine families. CX3CR1 is a specific receptor for the chemokine CX3CL1. CX3CL1-CX3CR1 plays a critical role in the anticancer immune response (33). Previous studies found that an increase in CX3CL1-CX3CR1 in tumor is associated with the forming of anti-cancer NK cells and CD8^+^T cells in tumor, which improves the prognosis for patients with gastric adenocarcinoma and glioma (34, 35). In this study, we investigated the anti-cancer effects of CX3CL1 in ccRCC. We found that CX3CL1 knockdown markedly promoted the migration and invasion of ccRCC cell *in vitro*. Targeted CX3CL1 therapy might provide new treatment directions for ccRCC patients.

Furthermore, molecular subtype analyses showed that the LR.score of the C3 subtype was low in both cohorts. Conversely, C1 showed higher scores in both cohorts, which is consistent with previous analyses and thereby confirms the effectiveness of LR-pairs. Meanwhile, in different cohorts, the prognostic model established using LR.score showed high validity and accuracy for the prognostic evaluation of patients. Furthermore, analysis of the differences in the LR.score between different clinicopathological features in two cohorts showed a significant association between the LR.score and patient’s malignancy grade. Therefore, LR.score can be used as a reliable biomarker for evaluating the prognosis of patients with ccRCC.

Infiltrating immune cells play various roles in different tumours, hence, the differences in immune score were assessed between patients in the different LR.score groups using ESTIMATE. The result showed that patients in the high-risk group showed higher immune score. A high immune score generally predicts a better prognosis, but patients in the high LR.score group showed a worse prognosis. However, the current findings are consistent with previous studies, revealing a significant positive correlation between immune score and malignancy degree in patients with ccRCC in the TCGA-KIRC cohort. High immune score and ESTIMATE scores have been associated with worse prognosis (36, 37). Additionally, the high infiltration levels of exhausted CD8^+^ T cells and immunosuppressive M2-like macrophages have been reported in advanced renal disease (8), suggesting that immune scores could indicate progressive T cell dysfunction in patients with ccRCC. This suggestion could also be used to explain a worse prognosis in patients with ccRCC that show a high immune score. Further, to explore the relationship between LR.score and immune cell infiltration levels, the immune cell infiltration levels of patients with ccRCC were quantified using CIBERSORT. Subsequently, the differences in the infiltration of 22 immune cells were compared, revealing that the CD8^+^ T cell infiltration level was significantly higher in the high LR.score group than in the low LR.score group. Previous studies have confirmed that the infiltration levels of CD8^+^ T cells are usually associated with a better prognosis in most solid tumours (38). Interestingly, the infiltration of CD8^+^ T cells was associated with a worse prognosis in ccRCC (39), which is consistent with the current findings. Therefore, the specificity of the TME of ccRCC can be accurately described using the LR.score model.

ccRCC is an immune-sensitive malignancy, and cytokine-based (IL-2 and IFN-α2b) regimens have been accepted for clinical use. Recent regimens that use immune checkpoints as a therapeutic modality have changed the treatment paradigm of ccRCC (40). However, a significant proportion of patients with kidney cancer do not respond to these therapies and those who initially respond show eventual tumour progression (41). Therefore, this study examined the relationship between immunotherapy and LR.score to assess the benefit of LR.score in different immunotherapy cohorts. The results showed that patients who responded to immunotherapy had significantly lower LR.score than those who responded less. Furthermore, the patients with higher LR.scores showed less favourable responses to immunotherapy. This suggests that single-agent immunotherapy could benefit patients with a lower LR.score. Additionally, the significant differences in survival between the high and low LR.score groups in both immunotherapy cohorts illustrate its association with immunotherapy.

Advances in high-throughput sequencing can lead to personalised therapeutics, wherein each patient’s cancer can be treated based on their genomic profile. Although high-throughput sequencing provides a large amount of genomic information, it requires professional bioinformatics analysis and interpretation. Moreover, linking key phenotypes or molecular reactions together remains challenging for most of the data. This study provides concise analysis and novel insights and directions for the personalized treatment of patients with ccRCC. Although high-throughput sequencing could provide a more refined research direction for disease treatment, the current cost of treatment remains high. Reducing treatment costs while maintaining treatment efficacy remains a challenge. Currently, there exists a large amount of research data that require accurate and effective analyses for better clinical application. Although the current study provides a clear view of cell types in the TME of ccRCC and increases our understanding of the importance of LR and cell-to-cell interactions in the microenvironment, there exist a few limitations. While multiple cohorts of patients with ccRCC were used as validation, clinical trial-based validation in larger ccRCC cohorts could better validate the current findings. Additionally, the current high-throughput sequencing data are based on transcriptome data. However, transcription levels are not necessarily associated with protein expression (42)since essential cellular functions are performed and regulated by the proteome, which is also worth considering. The interactions between adjacent cells are the basis of many biological processes, including signalling between cell ligand receptors. Current single-cell genome techniques analyse each cell individually after tissue dissociation, thus losing data on the location between cells. The LR interactions identified by the study may not occur when different cell types do not undergo spatial co-localization in the tumour, thus, spatial transcriptome analysis will help us further understand our findings. Various bioinformatics methods were used to simulate the cellular composition of TME in patients, and some conclusions are consistent with previous studies. However, in silico cell composition inevitably has limitations; for example, the cellular composition of TME cannot be fully displayed. Therefore, using multiple algorithms to simulate the composition of the patient’s TME simultaneously and conducting a more comprehensive analysis could better address the limitations of bioinformatic analyses.

In conclusion, the current study elucidates themicroenvironmental landscape of ccRCC, providing a comprehensive view of the cellular composition of TME in patients with ccRCC. The interaction and communication of LR-pairs between different cell types are evaluated while studying their cellular complexity in the TME. Two valuable LR-pairs models for the molecular subtyping of patients with ccRCC were identified based on LR-pairs in various cell types. Notably, this study provides new insights into cancer immunotherapy, where the expression patterns of LR-pairs effectively evaluate the prognosis of patients with ccRCC and are associated with the efficacy of immunotherapy. Therefore, the development of potential drugs targeting these LR-pairs could contribute to the clinical benefits of immunotherapy. This study provides a new direction in understanding TME and its clinical applications. It also provides novel ideas for identifying different tumour molecular subtypes and developing accurate and personalised tumour immunotherapy.

## Data Availability Statement

The datasets presented in this study can be found in online repositories. The names of the repository/repositories and accession number(s) can be found in the article/**Supplementary Material**.

## Author Contributions

QG, FL and PW designed the study. QG, FL, PW, YJ and WS conducted the study and analysed the data. FL drafted the manuscript. QG and YG revised the manuscript. All authors have read and approved the submitted version.

## Funding

This research was supported by the Guangxi Natural Science Foundation Project (No. 2020JJB140078).

## Conflict of Interest

The authors declare that the research was conducted in the absence of any commercial or financial relationships that could be construed as a potential conflict of interest.

## Publisher’s Note

All claims expressed in this article are solely those of the authors and do not necessarily represent those of their affiliated organizations, or those of the publisher, the editors and the reviewers. Any product that may be evaluated in this article, or claim that may be made by its manufacturer, is not guaranteed or endorsed by the publisher.

## Abbreviations

ccRCC, clear cell renal cell carcinoma; DEGs, differentially expressed genes; EGFR, epidermal growth factor receptor; GEO, Gene Expression Omnibus; HLA, human leukocyte antigen; GESA, gene set enrichment analysis; KEGG, Kyoto Encyclopedia of Genes and Genomes; KIRC, Kidney Renal Clear Cell Carcinoma; LR, ligand-receptor; MIF, macrophage migration inhibitory factor; PCs, principal components; RECA-EU, Research Concept and Research Activities- European Project; scRNA-seq, single-cell RNA sequencing; TCGA, The Cancer Genome Atlas; TGFβ, Transforming growth factor-beta; TME, tumour microenvironment; vSMC: vascular smooth muscle cells; WNT, wingless.
