# Supplementary figures and images for: Identification of Ligand-Receptor Pairs Associated With Tumour Characteristics in Clear Cell Renal Cell Carcinoma
Source: Front Immunol. 2022 Jun 6;13:874056. doi: 10.3389/fimmu.2022.874056 (PMC9207243; doi:10.3389/fimmu.2022.874056)

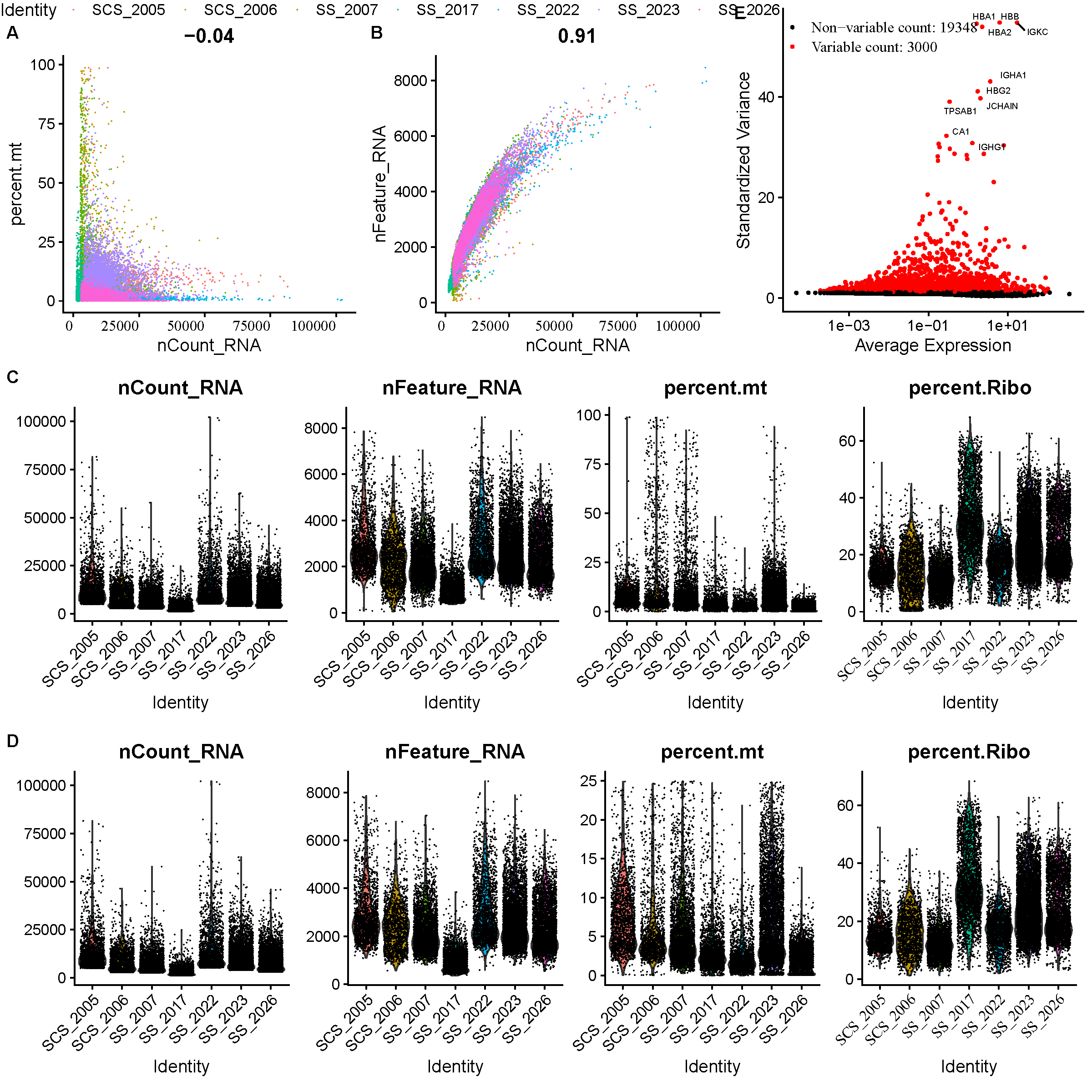

Supplement: Supplementary file 1 [file Image_1.tiff]

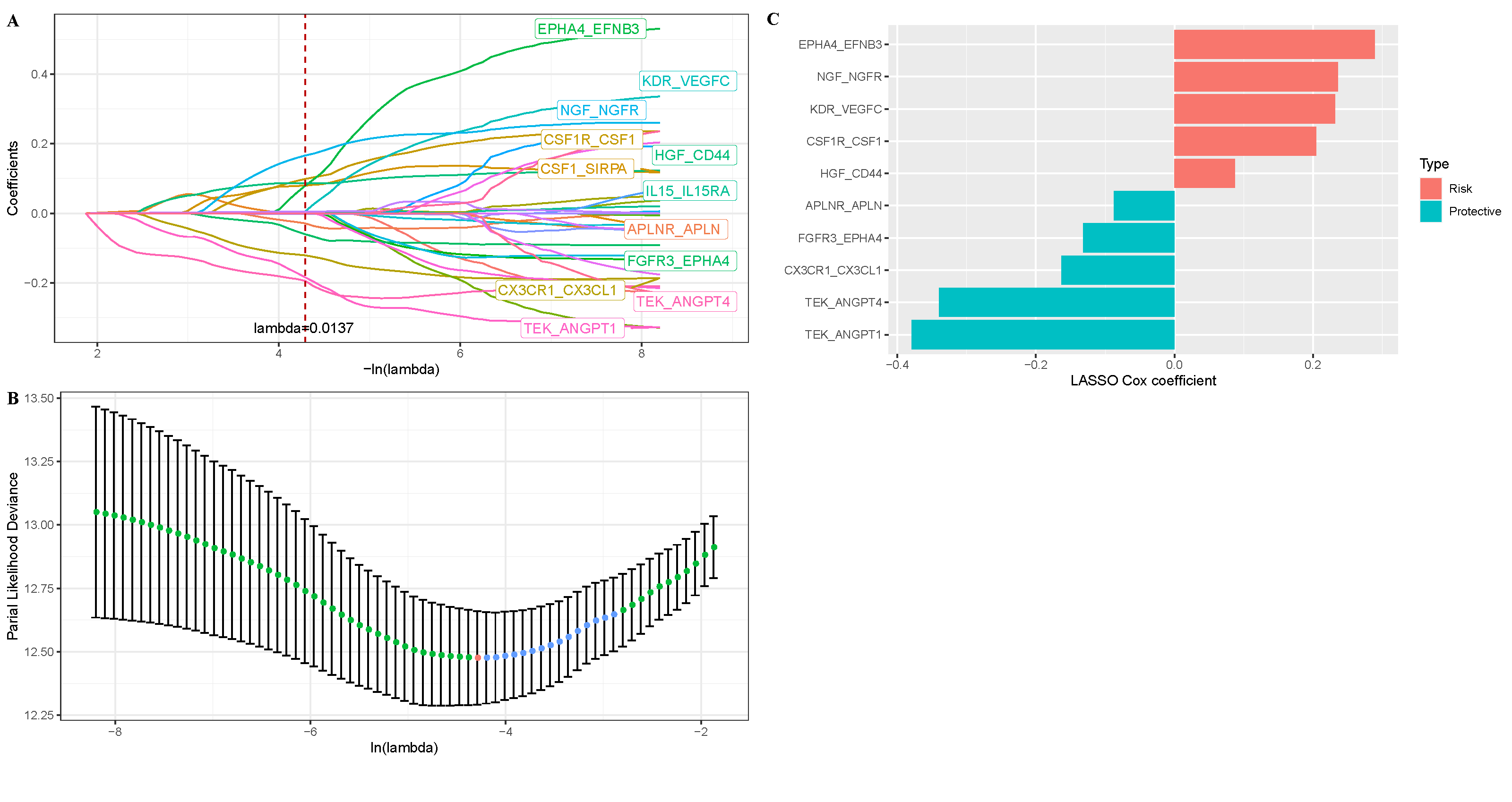

Supplement: Supplementary file 2 [file Image_2.tif]

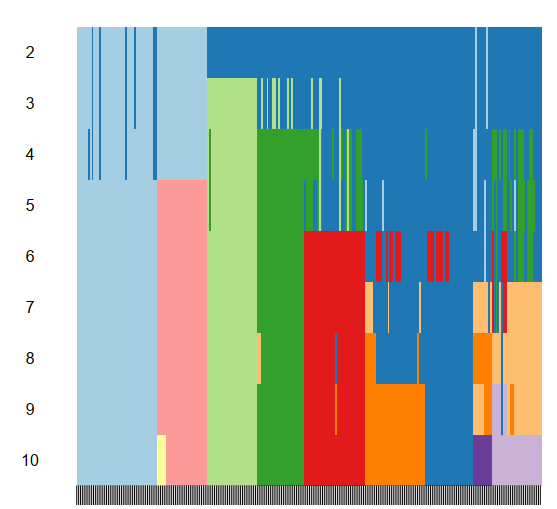

Supplement: Supplementary file 3 [file Image_3.png]
